# Open questions: what are the genes underlying antagonistic coevolution?

**DOI:** 10.1186/s12915-018-0583-7

**Published:** 2018-11-01

**Authors:** Dieter Ebert

**Affiliations:** Department of Environmental Sciences, University of Basel, Zoology, Vesalgasse 1, 4051 Basel, Switzerland

**Keywords:** Coevolution, Host–parasite interactions, Antagonistic coevolution, Selective sweeps, Negative frequency-dependent selection, Red queen dynamics

## Abstract

Although the idea of coevolution was first presented 150 years ago, we still only vaguely understand the genetic basis of its workings. Identifying the genes responsible for coevolutionary interactions would enable us to distinguish between fundamentally different models of coevolution and would represent a milestone in population genetics and genomics.

## Tackling the genetics of coevolution

As early as 1863, Charles Darwin had raised the idea of a coevolutionary model, suggesting that the unusual shape of the Madagascar Star orchid flower was the result of its long-term interactions with a highly specialized hawk moth. Since then, many biological phenomena have been ascribed to coevolution: exaggerated traits of offense and defense, sexual selection, biodiversity, and immune system evolution, reflected in the extraordinary genetic diversity of R-genes in plants and of the MHC in jawed vertebrates. The rationale for attributing these traits to coevolution stems from the idea of reciprocal adaptation: that changes in one species intensify selection on the antagonist species, and vice versa. This idea implies that change in one species is specific to biological features of the other species. These interactions thus lead to the high degree of specificity commonly observed in antagonistic interactions between hosts and parasites.

The best evidence for coevolution comes from studies on phenotypic changes, where one antagonist is tested, at different time points, in its interaction with isolates of the other antagonist, an approach successfully used in bacteria, animals, and plants [1–3]. While such time-shift experiments are powerful tools to detect coevolution, they generally do not reveal the genetic mechanism underlying the process. How many genes are involved in host–parasite interactions, and how are they organized in the genome? How do they interact, and how specific are these interactions? What form of selection operates on the genes? The genes and their mechanism of action have so far not been identified for a single case in nature, even though, in the last 50 years, a number of genetic models—both verbal and mathematical—have been put forth to describe the population genetic processes at work. These models, which describe an enormous diversity of coevolutionary scenarios [4–6], have shown that the coevolutionary process is highly dependent on, among other things, the genetic make-up of the populations, the source of genetic variation (mutations, gene flow, recombination), the size and structure of the coevolving populations, and the genetic architecture of the interacting genes and their effects for the phenotype. Earlier models focused on simple genetics with one or two loci while later models incorporated more loci or even assumed polygenic inheritance. From this diversity of models, it became clear that phenotypic assessments would only be able to identify the genetic mechanisms of coevolution in very simplified cases and that these assessments are very unlikely to hold up under natural conditions. Importantly, it has also become evident that it is not species that coevolve, but the genes and their associated phenotypes. This stresses the need for identifying the relevant genes to understand the mechanics of the coevolutionary process.

Two of the more frequently discussed genetic models are the selective sweep model and the Red Queen model [5]. Selective sweep coevolution is based on the idea that new mutations sweep to fixation in the populations of two coevolving species. Mutations may occur anywhere in the genome and increase in frequency, as long as they provide their bearer an advantage. Mutations do not need to alternate in the two populations. A population may have multiple mutations sweeping successively, and, in sexual organisms, multiple mutations may even sweep to fixation in different regions of the genome at the same time.

In contrast, the Red Queen model is based on a highly specific genetic architecture. It suggests that alleles at a few loci in the host and in the parasite respond differently to the antagonist, depending on the interacting genotypes. An allele *A* in the host may provide resistance to parasite type A, but susceptibility to parasite type B, while another allele (*B*) may do the inverse. This genetic architecture can prevent the fixation of alleles over evolutionary time scales. Because parasites track host alleles that cause susceptibility, a process of time-lagged negative frequency-dependent selection occurs, leading to cycles in allele frequencies. Over the long term, this process balances selection and maintains genetic variation at the disease loci. As alleles *A* and *B* can be maintained by balancing selection for long periods of time, they are likely to evolve, and selective sweeps may replace *A* with *A’* and *B* with *B′*. To make things more complicated, coevolution by selective sweep and by negative frequency-dependent selection may happen at the same time in different parts of the genome, as long as genetic recombination decouples their dynamics.

Although experimental and observational studies of phenotypes have reported indirect evidence for both the selective sweep and Red Queen models, it is difficult to infer the underlying genetic models from coevolving phenotypes. Indeed, given the complexity of naturally coevolving systems, it hardly seems possible. On the other hand, little direct genetic evidence exists. In a few cases, mutations (supposedly involved in coevolution) have been observed to spread in host or parasite populations, but not in the context of coevolution. And no case of cyclical allele frequency dynamics in association with disease has yet been observed in hosts and parasites. Thus, current support for the genetic models of coevolution is rather poor and mostly circumstantial. On the other hand, genome scans in diverse organisms uncovered that genomic regions presumably involved in host–parasite interactions stand out as being among the most rapidly evolving and most polymorphic genes in the genomes. This has led to intensive research into the causes and consequences of this diversity, even before a potential link to parasitic diseases was clear. It is now easy to find such regions even in non-model organisms, but in most cases, we can only speculate about the processes behind the observed patterns.

Answering the question “What are the genes underlying antagonistic coevolution?” would help us to overcome this shortcoming. To verify genetic models of coevolution, we need to find the genes in both antagonists, understand their interaction (function), and follow their temporal dynamics. With this information, we can place the study of coevolution into a population genetic framework. After all, genetic models of coevolution are firmly rooted in population genetics.

How can we find the genes underlying coevolution? Traditional approaches to finding genes associated with disease phenotypes use diverse forms of mapping panels, whole genome association frameworks, and proteomics. These approaches require good control of hosts and parasites, as separate approaches are usually necessary for the two antagonists. Exciting new developments allow for alternative approaches based on co-genomics—the simultaneous study of host and parasite genomes in order to locate genomic regions that show some form of association between the two antagonists [7–10]. These newly developed approaches enable us to uncover host genotype by parasite genotype interactions and associations with disease-related phenotypes with a higher precision than approaches based on only the host or the parasite genomes. Furthermore, it is possible to obtain snapshots of host–parasite associations from populations during their natural interactions [7].

## Why should biologists care about these questions?

Because infectious diseases are among the major threats to humans, livestock, and natural populations, the study of coevolution is not only of academic interest, but also of practical importance. By understanding the genetic architecture of coevolution, we can use population genetic models to predict such events as the expected temporal dynamics of allele frequencies, the likelihood of new mutations arising, and the spread of new variants. These insights would furthermore allow us to assess which factors—i.e., genetic recombination, mode of reproduction (sexual versus asexual), gene flow, population size, genome structure—are crucial in determining the course of coevolution. Currently these questions can only be addressed theoretically, with little empirical data.

Once we have identified the genes that underlie coevolution, new perspectives open up. Phylogenetic analysis provides us the tools to go deeper into the past to understand the origin of host–parasite coevolution and the timeframe over which it has developed. By understanding shared ancient polymorphisms of these genes, for example, we gain strong evidence for the long-term balancing selection that has acted upon them.

By identifying the genes underlying coevolution in multiple host–parasite systems, we also open the door to comparative analysis. Are coevolving genes host-, parasite- or system-specific? Are genes or genetic functions conserved? What types of genetic models for coevolution are found in different systems?

In conclusion, finding the genes behind antagonistic coevolution would help us understand the history, function, and evolution of these genes—a milestone in population genetics and genomics. We would not only understand the mechanism behind antagonistic coevolution, with all its implications for health, agriculture, and natural populations, we would also gain insights into the nature of the most diverse and rapidly evolving regions in our genomes, which have long been speculated to be the result of antagonistic coevolution.
